# Segmentation of 3D Point Clouds of Heritage Buildings Using Edge Detection and Supervoxel-Based Topology

**DOI:** 10.3390/s24134390

**Published:** 2024-07-06

**Authors:** Santiago Salamanca, Pilar Merchán, Alejandro Espacio, Emiliano Pérez, María José Merchán

**Affiliations:** 1 Departamento de Ingeniería Eléctrica, Electrónica y Automática, Escuela de Ingenierías Industriales, Universidad de Extremadura, Avda. Elvas s/n, 06006 Badajoz, Spain; ssalamanca@unex.es (S.S.); alespacior@unex.es (A.E.); 2 Departamento de Expresión Gráfica, Escuela de Ingenierías Industriales, Universidad de Extremadura, Avda. Elvas s/n, 06006 Badajoz, Spain; emilianoph@unex.es; 3 Departamento de Didáctica de las Ciencias Sociales, Lengua y Literatura, Facultad de Educación y Psicología, Universidad de Extremadura, Avda. Elvas s/n, 06006 Badajoz, Spain; mjmerchan@unex.es

**Keywords:** laser scanner, 3D point clouds, segmentation, heritage buildings, edge detection, supervoxels

## Abstract

This paper presents a novel segmentation algorithm specially developed for applications in 3D point clouds with high variability and noise, particularly suitable for heritage building 3D data. The method can be categorized within the segmentation procedures based on edge detection. In addition, it uses a graph-based topological structure generated from the supervoxelization of the 3D point clouds, which is used to make the closure of the edge points and to define the different segments. The algorithm provides a valuable tool for generating results that can be used in subsequent classification tasks and broader computer applications dealing with 3D point clouds. One of the characteristics of this segmentation method is that it is unsupervised, which makes it particularly advantageous for heritage applications where labelled data is scarce. It is also easily adaptable to different edge point detection and supervoxelization algorithms. Finally, the results show that the 3D data can be segmented into different architectural elements, which is important for further classification or recognition. Extensive testing on real data from historic buildings demonstrated the effectiveness of the method. The results show superior performance compared to three other segmentation methods, both globally and in the segmentation of planar and curved zones of historic buildings.

## 1. Introduction

3D point clouds in the construction industry have enabled the development of new applications that can significantly increase productivity and improve decision-making accuracy. These applications have reached the field of cultural heritage, helping the creation of Heritage/Historic Building Information Models (HBIMs) [1].

The creation of HBIMs begins with acquiring 3D data using laser scanners or photogrammetry, followed by data processing to generate parametric models. Currently, many processing tasks are manual due to complex surfaces and the lack of automated general procedures. A critical initial step is the structuring and basic interpretation of the raw 3D data. Simple geometric organization, like voxelization of the point cloud, is useful [2,3], but often insufficient because high-level information associated with the data is needed. Therefore, labelling data into meaningful classes is crucial, in addition to possible topological ordering, which is what voxels provide [4].

General solutions to the problem of 3D point cloud labelling can be categorized into two main groups [5]. The first involves the segmentation of the data followed by its classification. This is commonly referred to as pre-segmentation and classification/recognition algorithms. For the pre-segmentation phase, commonly used techniques include region growing [6,7,8,9], edge detection algorithms [10,11,12], or model fitting [13,14,15,16,17]. Classification often uses Machine Learning (ML) algorithms such as Support Vector Machine (SVM) [18] or Random Forest (RF) [9,19]. Recently, Deep-Learning (DL)-based methods have also been included [20,21,22]. The second group directly labels the raw 3D point cloud, primarily using ML and DL methods, with the 3D data as inputs and the labelled point cloud as outputs [23,24,25,26].

When the 3D point clouds are of historic structures, the problem becomes significantly more challenging. The scarcity of training datasets for neural networks limits the effectiveness of Deep Learning (DL) approaches [27]. Moreover, 3D data from heritage structures often have significant noise and uneven point densities due to the complex geometries and conditions of these sites. Lichti et al. [28] highlighted that the intricate details and material variability of heritage structures present challenges that increase noise levels. This complexity, together with the potential deterioration of heritage buildings, makes them more susceptible to noise than contemporary buildings with simpler geometries. Consequently, features from surface analysis used in ML, like those from principal component analysis (PCA), reflect this variability. Therefore, direct ML methods bypassing pre-segmentation may not be sufficiently effective [29].

This is why pre-segmentation and classification methods are advantageous in these scenarios. Region-growing is common for pre-segmentation, but its planarity assumption often leads to unsatisfactory results [7,30]. Model approximation methods tend to be more complex and may not work well when the data have significant variability or comprise different types of surfaces, so they are primarily used for plane segmentation [31,32]. Edge detection methods are less used due to their sensitivity to data variability, noise, and challenges with edge closure in unstructured point clouds [33]. However, in our opinion, edge detection algorithms have a great potential to produce good results. This is because they do not require a planarity hypothesis and can identify key elements of heritage structures (like columns, capitals, bases, arches, etc.) from their edges, which is essential for their subsequent classification. Therefore, these edge detection algorithms were used in this study.

In this paper, we propose a new unsupervised method designed to handle the challenges of processing cultural heritage 3D point clouds. Specifically, the segmentation procedure used a raw 3D point cloud and provided different parts that are significant from a heritage point of view. In order to solve the main problems of edge detection methods on 3D data, which are the sensitivity to data variability and noise, and the difficulty of edge closure, a new topological structure was proposed. This topological structure was used, in addition to the edge closure, for the final definition of the parts or segments of the point clouds. The proposed method was tested only on real data from historic buildings.

The paper’s main contributions are:

(a) Introducing a new unsupervised robust segmentation algorithm for 3D point clouds with high variability and noise, particularly suited for heritage building data.
(b) Formulating a procedure for the segmentation of 3D heritage data into distinct physical elements such as columns, capitals, vaults, etc. This segmentation, which is a per-object segmentation, allows for the precise identification of architectural features for further classification and analysis.
(c) Proposing a novel topological structure for 3D point clouds. Unlike common voxelization, this structure uses a graph that requires less computational memory and groups geometrically congruent 3D points in its nodes, regardless of graph resolution. This makes it highly effective for computer applications dealing with 3D point clouds.

The article is organized as follows. Section 2 briefly discusses the state of the art in segmentation methods. Section 3, Materials and Methods, is divided into two subsections. Section 3.1 describes the segmentation method and Section 3.2 explains the experimental setup. Section 4 shows the quantitative comparison of the results between our algorithm and three other methods. Finally, a discussion of the results is given in Section 5, and the conclusions of the work are explained in Section 6.

## 2. Related Works

The most commonly used procedures for heritage building 3D data are edge detection, model fitting, and region growing. Region-growing approaches utilize the topology and geometric features of the point cloud to group points with similar characteristics. Edge detection identifies the points that form the lines and edges. Model fitting involves approximating a parametric model to a set of points.

Although several of the processes mentioned are often used together, below is an analysis of several algorithms based on the most relevant segmentation procedure.

### 2.1. Region Growing

In region-growing algorithms [6], similitude conditions are applied to identify smoothly connected areas and merge them.

In Poux et al. [7], a region-growing method was used that starts from seed points chosen randomly to which new points are added according to the angle formed by their normals and the distance between them. This defines connected planar regions that are analyzed in a second phase to refine the definition of the points belonging to the edges. The main objective of the proposal is to avoid the definition of any parameters. Nonetheless, the results of the segmentation procedure are not entirely clear.

Huang et al. [9] proposed a different approach using the topological information generated after the first supervoxelization stage to merge these supervoxels according to flatness and local context. Clusters with different resolutions are obtained in the first stage. However, the results of the segmentation process are not presented, but those of the subsequent classification phase are, which is performed with a Random Forest classifier.

Zhang et al. [34] presented an unsupervised method for the semantic segmentation of 3D point clouds that starts with an initial supervoxelization (or superpoints) and progressively merges them in successive steps. To achieve this, they used a SparseConv neural network to determine the features of the cloud points, which are augmented with Point Feature Histograms (PFH) features. These features are used in a K-means algorithm to group the different points into increasingly larger supervoxels. The difference between the features of each point and the average features of each supervoxel are used to train the SparseConv network.

### 2.2. Edge Detection

When working with 3D point clouds with curved geometric elements, finding edge zones simplifies the segmentation problem since it is useful for delimiting regions of interest.

One of the first works on edge detection was proposed by Demarsin et al. [10]. The procedure starts with a pre-segmentation by region-growing based on the similarity between the normals. Next, a graph is created to perform edge closure. Finally, the graph is pruned to remove unwanted edges points. The algorithm requires very sharp edges to successfully detect the boundaries and it is very sensitive to the noise present in the data.

The Locally Convex Connected Patch (LCCP) [11,35] segmentation algorithm bases its performance on a connected net of patches which are then classified as edge patches and labelled either as convex or concave. Using the local information of the patches and applying several convexity criteria, the algorithm segments the 3D data without any training data. However, it is highly dependent on the parameters used to apply the criteria.

More recently, Corsia et al. [12] performed a shape analysis based on normal vector deviations. The detected edge points allow the posterior region-growing process to have a coherent oversegmentation in complex industrial environments.

### 2.3. Model Fitting

Model fitting methods are usually based either on Hough Transform (HT) [36] or on Random Sample Consensus (RANSAC) [37].

The Constrained Planar Cut (CPC) [14] method advances LCCP [11,35] by incorporating a locally constrained and directionally weighted RANSAC from its initial stages, which improves edge definition to segmentate 3D point clouds into functional parts. This technique enables more accurate segmentation, especially in noisy conditions, by optimizing the intersection planes within the point cloud. Since CPC uses a weighted version of RANSAC, it has even more parameters to adjust than LCCP, complicating the configuration for non-conventional point clouds.

Macher et al. presented a semi-automatic method for 3D point cloud segmentation for HBIMs [15]. This algorithm is RANSAC-based and capable of accurately detecting geometric primitives. However, it requires the significant manual adjustment of parameters to accommodate different building geometries, which raises issues of scalability and ease-of-use across diverse datasets. In addition, no numerical results were presented to allow rigorous analysis of the results. The procedure proposed in [30] extracts planar regions by using an extended version of RANSAC and applies a recursive global energy optimization to curved regions to achieve accurate model fitting results, but large missing areas in the dataset lead to oversegmentation.

Other approaches exist for planar region extraction, apart from RANSAC. Luo et al. [16] used a deterministic method [17] to detect planes in noisy and unorganized 3D indoor scenes. After the planar extraction task, this approach examines the normalized distance between patches and surfaces before implementing a multi-constraint approach to a structured graph. In case this distance is not sufficient to split the objects, color information is also used. The segmentation results of indoor 3D point clouds are good, but thanks to the use of the sensory fusion technique described.

## 3. Materials and Methods

### 3.1. Segmentation Method

#### 3.1.1. Overview of the Segmentation Method

**Figure 1:**
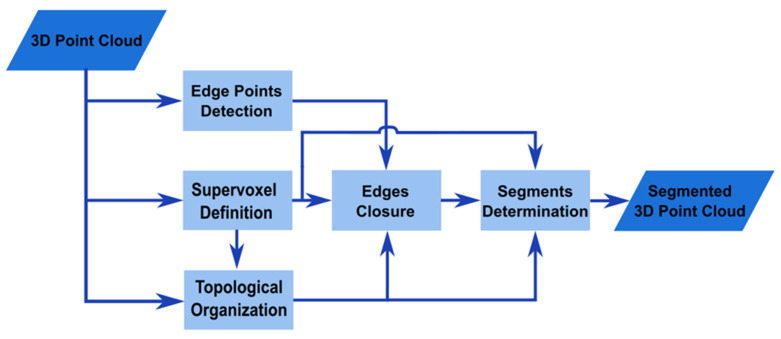
Diagram of the procedure followed for edge-detection-based segmentation of 3D point clouds of heritage buildings.

The outline of the segmentation method for 3D point clouds of heritage buildings presented in this paper is shown in Figure 1.

The input to the algorithm is the 3D data, which was acquired with laser scanners, photogrammetry, or other equivalent technologies.

First, the edge points are detected and labelled. To avoid false negatives, a conservative algorithm is employed at this stage. This algorithm is explained in Section 3.1.2.

In parallel, a supervoxelization method is applied to the complete 3D point cloud. After that, a graph-like topological structure is created using the supervoxels and the 3D points. These two processes are detailed in Section 3.1.3.

The previously computed elements are used in the next phase to generate the closure of all edges. This step also identifies edge-supervoxels. This is dealt with in Section 3.1.4.

Finally, a segmentation algorithm is applied. Starting from a non-edge-supervoxel, an assignation process, following the topological structure defined by the graph, is constructed. We discuss this issue in Section 3.1.5.

#### 3.1.2. Edge Point Detection

The main edge detection methods for 3D point clouds are based on curvature [38,39] or normal values [40,41].

However, both features tend to suffer from data with high variability. Therefore, we decided to use the edge detection algorithm proposed by Ahmed et al. [42], which uses neither curvature nor normals.

Formally, a 3D point, $p_{i}$, belongs to an edge if the following is verified:

(1) $$\frac{\left\|C_{i}-p_{i}\right\|}{Z_{i}\left(V_{i}\right)}<\gamma ,$$

where $V_{i}$ is the set of k neighbors of $p_{i}$, $C_{i}$ is the centroid of $V_{i}$, $Z_{i}\left(V_{i}\right)$ is the minimum distance from $p_{i}$ to a 3D point of $V_{i}$, and γ is a parameter that defines the classification threshold.

If a point $p_{i}$ satisfies condition (1), it will be labelled as an edge point if there are five other edge points in its vicinity. This eliminates the false positives in the algorithm.

In a point cloud $N=\{p_{1},\;p_{2},\;\ldots ,\;p_{n}\}$, we denote the edge point set as $B_{N}$.

#### 3.1.3. Supervoxelization and Topological Organization

The supervoxelization of 3D data is a natural extension of superpixel detection in 2D images. These methods divide the 3D point cloud into meaningful regions, with the characteristic that this is an oversegmentation of the data. Oversegmentation reduces the complexity of post-processing while preserving essential structural and spatial information, being a crucial step in many computer vision tasks [43,44].

Among all the methods proposed for the supervoxelization of 3D point clouds, we used the algorithm discussed in [45], since it is an edge-preserving algorithm. This feature proves vital when the proposed segmentation method relies on an edge point detection algorithm.

This method initially considers each 3D point to be a supervoxel. Iteratively, nearby supervoxels will be clustered following a minimization procedure of an energy function, $E\left(Z\right)$, occurring whenever the following condition is satisfied:

(2) $$\lambda -c_{j}*D\left(r_{j},r_{i}\right)>0,$$

where λ>0 is a regularization parameter that initially takes a small value and increases iteratively; $c_{j}$ is the number of points in the supervoxel $S_{j}$; and $D\left(r_{j},r_{i}\right)$ is a distance metric between the centroids, $r_{j}$ and $r_{i}$, of the candidate supervoxels to be joined, $S_{j}$ and $S_{i}$. This metric is defined as follows:

(3) $$D\left(r_{j},r_{i}\right)=1-\left|n_{j}\cdot n_{i}\right|+0.4\cdot \frac{∥r_{j}-r_{i}∥}{R},$$

where $n_{j}$ and $n_{i}$ are, respectively, the normal vector associated with the supervoxels $S_{j}$ and $S_{i}$, and R is the resolution of supervoxels.

Once the algorithm is completed, the set of k supervoxels of the point cloud, $N=\left\{p_{1},p_{2},\ldots ,p_{n}\right\}$, denoted as $S_{N}=\{S_{1},S_{2},\ldots ,S_{k}\}$, is obtained, where k<n. $S_{j}=\left\{p_{i}^{S_{j}},\forall i\in \left\{1,\ldots ,k\right\}|p_{i}^{S_{j}}\in N\right\}$ is the set that stores the points of N that form the supervoxel j.

Formally, the algorithm implements a non-injective surjective function, that we called the supervoxel assignation function, $s:\{p_{1},p_{2},\ldots ,p_{n}\}->\{S_{1},S_{2},\;\ldots ,S_{k}\}$, in which each 3D point $p_{i}\in N$ is associated with a supervoxel of N. The inverse set-valued function, called the point assignation function, $s^{-1}\left(\cdot \right)$, also exists, and it is determined by the algorithm. The condition k<n ensures that there are more points in the 3D cloud than supervoxels.

The following step involves constructing a graph structure, $G_{N}$, that organizes the point cloud topologically, facilitating the remaining segmentation process. In $G_{N}$, each node corresponds to one of the supervoxels of the point cloud.

To establish the edges of $G_{N}$, we first identify the 3D points that lie on the boundaries between the different supervoxels. Then, we connect the supervoxels in the graph whose boundary points touch each other. This connection forms the edges of the graph, thus connecting adjacent supervoxels.

To do this, we start by looking for the q nodes belonging to a given supervoxel $S_{i}$, using the point assignation function, $s^{-1}\left(S_{i}\right)$. Next, we search for the nearest k neighbors to each of the $p_{j}^{S_{i}}\in S_{i}$ using a k-NN search [46]. This set of adjoints pointed to $p_{j}^{S_{i}}$ is denoted as $A\left(p_{j}^{S_{i}}\right)=\left\{p_{a_{1}^{j}},\;p_{a_{2}^{j}},\ldots ,\;p_{a_{m}^{j}}\right\}$. Next, the supervoxel assignation function, $s\left(p_{a_{k}^{j}}\right)$, is applied $\forall \;p_{a_{k}^{j}}\in A\left(p_{j}\right)$, to obtain the supervoxel to which the points in $A\left(p_{j}\right)$ belong.

From this set, we define a logical function $B_{S_{i}}\left(p_{a_{k}^{j}\;}\right)$ that returns TRUE if the point $p_{a_{k}^{j}\;}$ is on the edge of the supervoxel $S_{i}$, and FALSE otherwise, according to the following definition:

(4) $$B_{S_{i}}\left(p_{a_{k}^{j}}\right)=\left\{\begin{array}{cc} \mathrm{TRUE}, & \mathrm{if}\;\;s\left(p_{a_{k}^{j}}\;\right)-S_{i}\neq \emptyset \\ \mathrm{FALSE}, & \mathrm{Otherwise} \end{array}\right.,$$

The set difference operation $s\left(p_{a_{k}^{j}}\;\right)-S_{i}$ gives us a subset of the supervoxels that are neighbors of the supervoxel $S_{i}$ and are close to the point $p_{a_{k}^{j}\;}$. If we apply the function $s\left(p_{a_{k}^{j}}\;\right)$ to all points in the supervoxel, perform the union operation on these points, and then subtract $S_{i}$, we obtain the set of neighbors of $S_{i}$.

Formally, the set of supervoxels neighboring $S_{i}$, that is denoted as $NS_{i}$, are given by the following expression:

(5) $$NS_{i}=\left({⋃}_{j=1}^{q}{⋃}_{k=1}^{m}s\left(p_{a_{k}^{j}}\;\right)\right)-S_{i},$$

In conclusion, $G_{N}$ is defined based on the k supervoxels and the neighboring supervoxels of each supervoxel. This results in a graph that encapsulates the topological organization of the point cloud. Mathematically, we can define the vertices and edges of $G_{N}$ as follows:

**Definition** **1.** *Let* V* be the set of vertices and * E *be the set of edges in the graph* $G_{N}$ *. Then:*

$V=\{S_{1},\;S_{2},\;\ldots ,S_{k}\}$*, where each* $S_{i}$ *is a supervoxel.*
$E=\left\{\left(S_{i},S_{j}\right)|S_{j}\in NS_{i}⋁S_{i}\in NS_{j},\forall i,j\in \left\{1,2,...,k\right\}\right\}$, *where* $NS_{k}$ *is the set of neighboring supervoxels to* $S_{k}$.

Our graph is an undirected type, which means that an edge between the two vertices $S_{i}$ and $S_{j}$ is identical to an edge between $S_{j}$ and $S_{i}$. This characteristic is reflected in our definition of the set of edges, E.

#### 3.1.4. Edge Closure

From $B_{N}$ and $S_{N}$, we obtain the set of edge-supervoxels, $S_{B}$, which are the ones where the edge closure of N is achieved.

A supervoxel can be considered an edge-supervoxel if it contains at least one edge point. Formally,

**Definition** **2.** *Let* $S_{B}$ *be the set of edge-supervoxels. Then,* $S_{B}=\left\{S_{j}|\exists \;p_{i}\in S_{j}⋀p_{i}\in B_{N}\right\}$.

#### 3.1.5. Segment Determination

The last stage of the algorithm consists of determining the different regions of the point cloud. To do this, we perform two steps:

1. Region growing of supervoxels from a seed supervoxel not belonging to $S_{B}$.
2. Inclusion of edge-supervoxels in one of the regions identified in step 1.

The region-growing algorithm does not make use of geometrical similarity analysis between supervoxels, only the set of supervoxels, $S_{N}$, and the topological sorting provided by G.

If we denote the different regions as $R=\{R_{1},\;R_{2},\ldots \}$, and assuming that we are creating the region $R_{k}$, this step can be performed by following the following steps:

1. Initialize $R_{k}=\emptyset$
2. Choose a supervoxel $S_{k}\notin \;S_{B}$ not yet assigned to another region.
3. ${R_{k}=R_{k}⋃S}_{k}$.
4. Determine the edges of $S_{k}$ in G, $E(S_{k})=\{S_{i},\;S_{n},\;S_{l},\;\ldots \}$.
5. $R_{k}=R_{k}\cup S_{j}$, iff $S_{j}\in E\left(S_{k}\right)$ and $S_{j}\notin S_{b}$.
6. Choose as new $S_{k}$ a supervoxel of $R_{k}$ of which the neighborhood in the network has not yet been analyzed.

If there is $S_{k}$ that satisfies the condition in step 6, go back to step 4. Otherwise, the algorithm is finished.

This algorithm is repeated until every non-edge-supervoxel is assigned to a region.

The final stage of segmentation is the inclusion of edge-supervoxels in the regions identified previously. For this purpose, we will analyze the edge-supervoxels, according to G, and divide them into the following three types:

- Edge-supervoxels that have some non-edge-supervoxel neighbors and all of them belong to a unique region. In this case, the supervoxel in question is assigned to the region to which its neighbors belong.
- Edge-supervoxels that have some non-edge-supervoxel neighbors belonging to different regions. In this case, we apply Equation (2) and assign the edge-supervoxel to the region of the supervoxel with a lower distance value.
- Edge-supervoxels in which all its neighbors are edge-supervoxels. The edge-supervoxel is not assigned yet.

After applying these rules, some edge-supervoxels may not be assigned to any region. Therefore, the procedure must be repeated iteratively until all supervoxels are assigned.

### 3.2. Experimental Setup

To verify the validity of the proposed method, the algorithm was programmed in MATLAB©, while the supervoxelization algorithm and the comparison methods were performed using C++. Comparison results are presented in Section 5.

Each experiment was run on an AMD Ryzen 7 5800X 8-core 3.80 GHz CPU with 32 GB of RAM.

#### 3.2.1. Point Cloud Dataset

We tested the method in five different 3D point clouds. We used LiDAR point clouds from our repository and part of the ArCH dataset from [47].

Point clouds from our repository (PC1, PC2, and PC3) were acquired using the Leica BLK360 scanner, while point clouds from the ArCH dataset (PC4 and PC5) were acquired using TLS and TLS + UAV.

We down-sampled each point cloud for time-saving purposes since we developed our method using the MATLAB framework.

**Figure 2:**
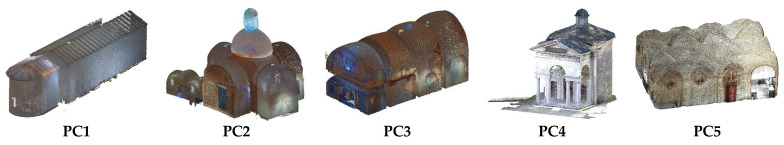
RGB point clouds for testing purposes.

The characteristics of these point clouds are listed in Table 1, where the dimensions of the point cloud refer to the size of the Best Bounding Box of the point cloud. For better visualization, Figure 2 shows the point clouds in their true color.

#### 3.2.2. Algorithm Parameter Values

There are only 3 parameters to set in the algorithm: the threshold for edge point classification, γ (Section 3.1.2); the resolution of the supervoxels, R (Section 3.1.3); and the number of neighbors of each point in the graph generation phase, k (Section 3.1.2 and Section 3.1.3).

Regarding γ, the value used is the one proposed by the authors of [42], which is 0.5. For a given point cloud, regardless of its point density, the lower the value of R, the more supervoxels will be generated. However, the larger the size of the heritage building is, the higher the value of R should be. In our case, we used a value of 0.1 m, which provides a good resolution for all point clouds without heavily increasing the number of supervoxels, deteriorating the method’s performance.

Finally, for the detection of border points and the definition of the graph, G, it is necessary to define k, which for both cases is 50.

#### 3.2.3. Accuracy Evaluation Metrics

To quantitatively compare our method, we calculated the parameters Precision (6); Recall (7); F1-score (8); and Intersection over Union (9).

(6) $$Pe\;\;=\;\frac{TP}{TP\;+\;FP},$$

(7) $$Re\;=\;\frac{TP}{TP\;+\;FN},$$

(8) $$F1\;=\;\frac{2\cdot Pe\cdot Re}{Pe+Re},$$

(9) $$IoU\;=\;\frac{TP}{TP+FP+FN},$$

In these equations, TPs are the True Positives, FPs the False Positives, and FNs the number of False Negatives. These parameters are calculated by comparing the segments defined in the ground truth versions of the point clouds with the segments obtained from Locally Convex Connected Patches (LCCPs) [11,35], Constrained Planar Cuts (CPCs) [14], and the Region-Growing (RG) algorithm of Rabani et al. [6], and our algorithm (for simplicity, we will refer to it as ‘Ours’ from now on).

Once these values have been calculated, the parameters Pe, Re, F1, and IoU are calculated for the entire point cloud to get an idea of how the segmentation works globally. However, since our main goal is to segment unconventional and historical buildings, we will also perform a second study that discriminates between flat and curved regions to compare the performance in different types of areas.

## 4. Results

### 4.1. Global Results

Table 2 shows the parameters calculated globally, i.e., for each of the meshes as a single entity. In order to better compare the segmentation results, the highest F1 or IoU value for each of the point clouds is shown in bold. As can be seen, the algorithm presented in this paper gives the best result in 100% of the cases, proving the validity of the method.

**Figure 3:**
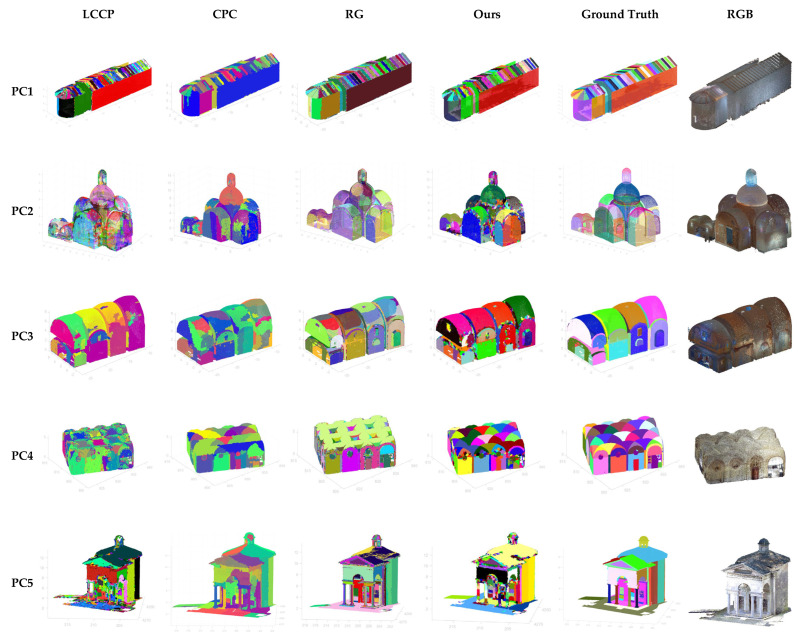
Visual comparison between segmentation results and ground truth.

Figure 3 shows the visual comparison between methods for each point cloud where each color represents a different segment.

### 4.2. Curved and Planar Segment Results

We manually differentiated between plane and curved regions in the ground truth version of each point cloud and recalculated the evaluation metrics. Table 3 and Table 4 show these results. As with the global results, the best result for F1 and IoU is shown in bold.

In the case of flat segments, the results show the following:

1. The F1 parameter of our algorithm was the best in 60% of the results. This means that in most cases, the proposed method provided a segmentation of flat areas with a maximum number of TPs without a significant number of FPs and TNs. The method we called RG was the second-best method according to the F1 parameter.
2. Taking into account the IoU parameter, the algorithm with the best results in 60% of the cases was RG. Therefore, the method that best aligned the predicted segments spatially with the real ones, which is what IoU measures, was RG. The second-best method according to this parameter was the one presented in this paper.

The good performance of the RG algorithm on flat surfaces was as expected, as it was primarily designed for plane segmentation. Nevertheless, the method presented in this paper showed equivalent results to those of RG.

In the case of curved segments, our algorithm once again gave the best results in 80% of the cases, both for *F1* and for *IoU*.

**Figure 4:**
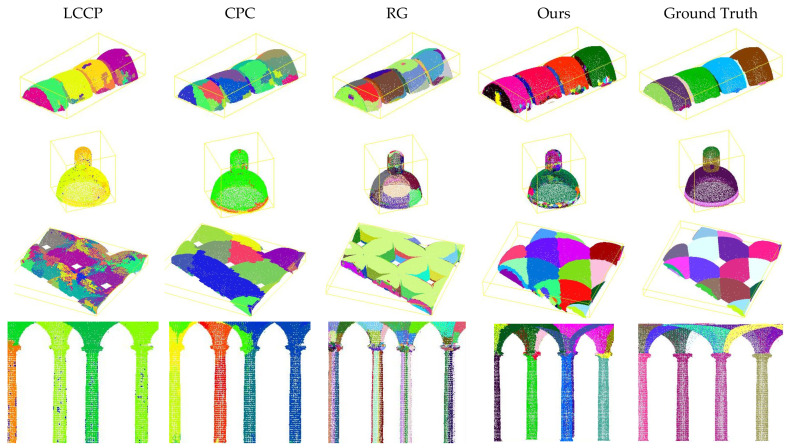
Visual comparison of heritage architectural elements.

Figure 4 focuses on showing the similarities between segmented regions of architectural elements from our method and the ground truth.

## 5. Discussion

### 5.1. Strengths

The results section shows that our method significantly improved the tested methods’ results when applied to 3D point clouds from heritage buildings. The proposed method is completely unsupervised, i.e., no prior learning process with labelled data is required, which is essential in the case of applications with 3D cultural heritage data, where there is no significant amount of labelled data.

The edge closure procedure overcame one of the major problems of 3D data segmentation methods based on edge detection. Our proposal is independent of both the edge detection procedure and the supervoxelization method, so it can be easily adapted to other algorithms that solve these problems.

Thanks to the proposed edge detection process, our method detects smoother changes and thus achieved a better delimitation for the region-growing step, which allows us to successfully segment elements of the architectural heritages, which usually present gradual normal vector variations. This is shown in Figure 4, which also demonstrates that our method was the only one that successfully segmented, for example, the different domes of the point clouds PC2, PC3 and PC4, as well as almost all the constituent elements of the dome of PC2.

Although this algorithm performed best in curved areas, it still produced good results when segmenting planar parts, being similar to the results of segmentation methods based on plane detection [6].

The storage size of the presented topological structure was improved compared to the most common structure based on the voxelization of 3D points. Furthermore, it can be considered as a mesh over the 3D points and can be used as a multi-resolution structured representation of the 3D point clouds. The resolution of the graph depends on the value of R chosen for the supervoxel, which makes the graph useful as a framework for other algorithms such as semi-supervised segmentation methods using graph neural networks [46]. This may be one of the most interesting lines of work today for working with weakly labelled data.

### 5.2. Limitations and Research Directions

Minor details in heritage point clouds, such as some moldings and columns, may be merged into larger supervoxels or divided into different regions. The dome element shown in Figure 4 demonstrates how our method detects the molding under the main area, although it was divided into several segments because many points are detected as edge points.

The more the value of R increases, the less accurate the method becomes, due to the larger size of the supervoxels. It is therefore necessary, if possible, to determine the most appropriate value of R for the case in question.

Finally, it should be noted that the method presents execution times in the order of hours, which may be a high execution time depending on the subsequent application of the performed segmentation.

## 6. Conclusions

Segmenting point clouds from heritage buildings is a challenging task due to their non-uniform density and distribution of points, as well as the high variability of the data employed. This paper proposes a new segmentation method for high-variability 3D point clouds of heritage and non-conventional buildings, which output great results.

By mixing edge detection, supervoxelization, and a new graph-based topological structure on 3D points, we developed a robust algorithm capable of accurately segmenting architectural elements in historical point clouds.

Our method outperformed the tested methods in this paper, with great results particularly when applied to curved zones. Although the method may output some errors, the overall quality and accurate sub-segmentation rate demonstrate that this method is suitable to be tested in subsequent classification tasks.

## Figures and Tables

**Table 1:** Characteristics of point clouds used for testing purposes.

Point Cloud	N° of Points	Length (m)	Width (m)	Height (m)
PC1	486,937	26.7	6.86	7.34
PC2	218,647	18	22.83	16.83
PC3	282,870	17.73	10.91	8.47
PC4	908,122	18.82	16.36	5.73
PC5	825,088	17.4	17.58	13.57

**Table 2:** Global quantitative analysis results.

Point Cloud	LCCP				CPC				RG				Ours			
	Pe	Re	F1	IoU	Pe	Re	F1	IoU	Pe	Re	F1	IoU	Pe	Re	F1	IoU
PC1	0.685	0.530	0.598	0.271	0.636	0.540	0.584	0.413	0.840	0.670	0.746	0.594	0.889	0.752	0.815	0.688
PC2	0.651	0.387	0.486	0.413	0.574	0.466	0.514	0.346	0.840	0.617	0.711	0.552	0.920	0.618	0.740	0.587
PC3	0.684	0.552	0.611	0.288	0.668	0.549	0.602	0.431	0.920	0.703	0.797	0.662	0.929	0.791	0.854	0.745
PC4	0.521	0.494	0.507	0.123	0.536	0.403	0.460	0.299	0.669	0.452	0.540	0.370	0.856	0.601	0.706	0.545
PC5	0.449	0.287	0.346	0.519	0.475	0.390	0.428	0.273	0.713	0.507	0.592	0.421	0.870	0.604	0.731	0.576

**Table 3:** Quantitative analysis results for plane regions.

Point Cloud	LCCP				CPC				RG				Ours			
	Pe	Re	F1	IoU	Pe	Re	F1	IoU	Pe	Re	F1	IoU	Pe	Re	F1	IoU
PC1	0.619	0.490	0.547	0.376	0.651	0.575	0.611	0.440	0.881	0.727	0.797	0.662	0.963	0.869	0.913	0.841
PC2	0.758	0.374	0.501	0.334	0.683	0.431	0.529	0.359	0.894	0.811	0.850	0.739	0.937	0.798	0.862	0.724
PC3	0.684	0.648	0.666	0.499	0.728	0.585	0.649	0.480	0.941	0.892	0.916	0.845	0.925	0.886	0.905	0.826
PC4	0.550	0.568	0.559	0.388	0.612	0.412	0.492	0.327	0.870	0.752	0.807	0.676	0.918	0.510	0.656	0.488
PC5	0.437	0.272	0.336	0.202	0.475	0.390	0.428	0.273	0.708	0.533	0.608	0.436	0.883	0.698	0.779	0.638

**Table 4:** Quantitative analysis results for curved regions.

Point Cloud	LCCP				CPC				RG				Ours			
	Pe	Re	F1	IoU	Pe	Re	F1	IoU	Pe	Re	F1	IoU	Pe	Re	F1	IoU
PC1	0.476	0.264	0.334	0.205	0.429	0.241	0.309	0.183	0.522	0.329	0.404	0.258	0.550	0.362	0.437	0.280
PC2	0.549	0.406	0.467	0.305	0.504	0.384	0.436	0.279	0.699	0.343	0.460	0.299	0.854	0.492	0.633	0.463
PC3	0.684	0.367	0.477	0.314	0.574	0.489	0.528	0.359	0.845	0.398	0.542	0.377	0.938	0.642	0.762	0.615
PC4	0.451	0.361	0.402	0.252	0.445	0.389	0.415	0.262	0.238	0.110	0.150	0.081	0.799	0.739	0.768	0.623
PC5	0.501	0.340	0.404	0.253	0.622	0.446	0.520	0.260	0.764	0.363	0.492	0.326	0.608	0.326	0.457	0.296

## Data Availability

Dataset available on request from the authors.
